# Fifty-five years in sleep research: contributions, experiences, and progress

**DOI:** 10.1093/sleepadvances/zpad021

**Published:** 2023-06-07

**Authors:** Merrill M Mitler

**Affiliations:** Commonwealth Health Research Board, Richmond, VA, USA

This paper is part of the Living Legends in Sleep Research series, which is sponsored by Idorsia Pharmaceuticals and Jazz Pharmaceuticals.

This paper is respectfully submitted in response to an invitation from the Editors of *SLEEP Advances*. From the several options offered to me, I chose to give a personal perspective that begins with my studies at Michigan State University and continues with my sleep research at Stanford University, Stony Brook University/Long Island Research Institute and The Scripps Clinic and Research Foundation. I was also asked to summarize some of the most significant aspects of my government work pertaining to sleep disorders at the National Institutes of Health in Washington. Since the overall timeframe is a bit over 55 years, readers must excuse a few gaps in the chronology. Also excluded from this narrative is my work from 2016 to the present as Scientific Consultant with The Commonwealth Health Research Board, an agency which provides financial support by means of competitive grants for human health research in the Commonwealth of Virginia [https://www.chrb.org/about.shtml].

I trained as a developmental psychologist. An emphasis in my training was the measurement of change over developmental time in behaviors and abilities. One lesson from my training that I will not forget is that an investigator must always *inspect* data in an unprocessed state to look for errors and unanticipated patterns. Then the investigator must proceed with the preplanned statistical analyses. However, the investigator is free to perform post hoc analyses including those that may be suggested from inspection of data. One central idea of inspection is that the human eye can detect patterns and trends that often are missed by number crunching processes. This lesson has proven to be crucial in my career.

Being in Developmental Psychology, as I was, I got hooked on sleep research by a 1966 paper in Science, “Ontogenetic development of the human sleep-dream cycle” [1]. Here, Roffwarg et al. proposed that CNS development in young animals was aided by endogenous sensory stimulation during REM sleep. The more immature the animal is, the less the animal can obtain sensory stimulation from interacting with its environment. The general idea is that an immature CNS needs more endogenous stimulation than a mature CNS, hence more REM sleep. This notion fit with findings in the literature of more REM sleep in premature human infants than in full-term infants. The idea also fit with data from various species that varied in CNS maturity at the time of birth.

I was told that Danielle Jouvet-Mounier in Michel Jouvet’s laboratory in Lyon, France was developing methods of surgically implanting recording electrodes in newborn and pre-born rodents to track sleep stages in early development [2, 3]. In 1968, on the advice of my doctoral thesis advisor, Ralph Levine, I travelled to Lyon to visit and learn. The Jouvets were extremely helpful, and I eventually became immersed in the field.

After completing my PhD at Michigan State University, the most important career choice I made was on the advice of Michel Jouvet and Allan Rechtschaffen to accept a post-doctoral fellowship with William C. Dement (Bill) at Stanford University Medical School. I arrived at Stanford in 1970. Here, I joined a diverse group of physicians, neuroscientists, graduate students, and undergraduate students working with Bill on multiple independent projects involving sleep and sleep disorders. As mentioned earlier, quantitative characterization of signs and symptoms in children and experimental animals was a key aspect of my training. That training proved useful as this era of sleep medicine unfolded.

## Cataplexy in Cats

**Figure 1.**
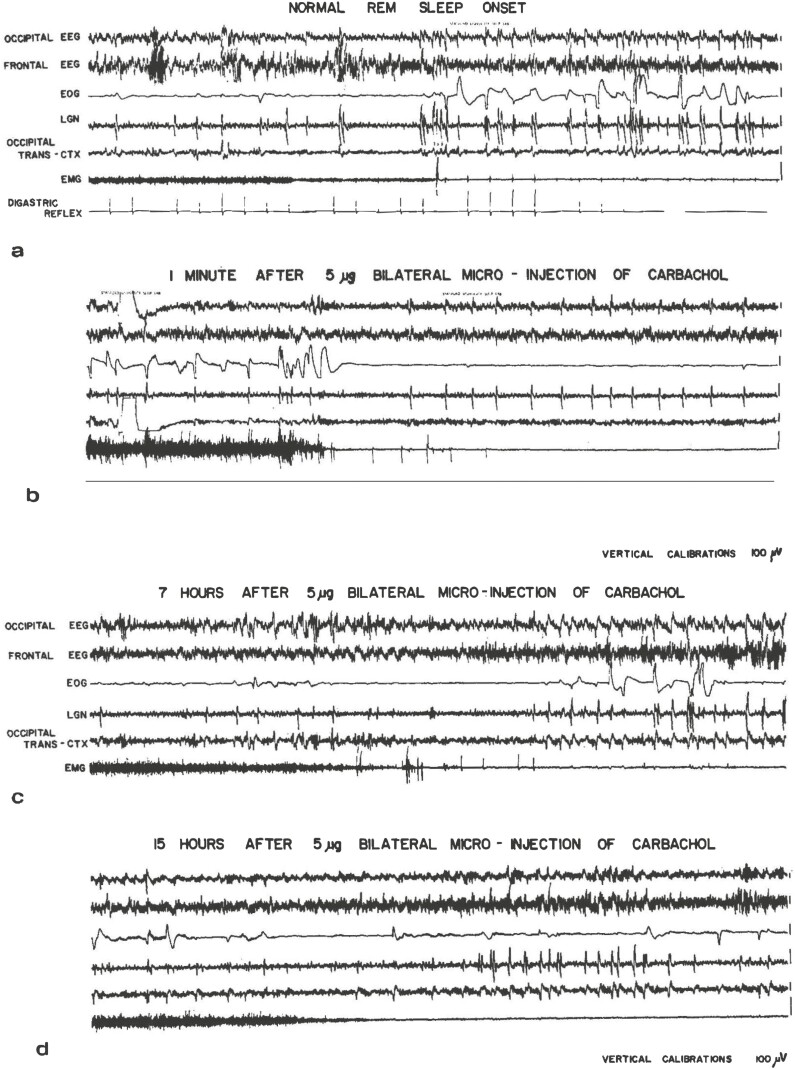
Effects of bilateral micro-injections of carbachol (reprinted from [4] with permission) These 30-second tracings represent the effects of bilateral micro-injections of carbachol 5 micrograms of carbamylcholine chloride in the pontine reticular formation of chronically implanted cats. a: baseline; b: early response to injection; c: representative episode of atonia 7 hours post-injection; d: representative episode of atonia 15 hours post injection. Note the eye movement and PGO activity during atonic periods.

At Stanford, Bill had just founded the first sleep disorders clinic based on a fee-for-service medical model. The Stanford Sleep Clinic began diagnosing and treating numerous patients with symptoms of excessive sleepiness severe enough to interfere with their livelihoods. Prominent among such patients were those found to have narcolepsy. The derangement of REM sleep mechanisms in narcolepsy suggested to us that brainstem cholinergic systems were inappropriately activated during sleep attacks and cataplexy. With the encouragement of Barry Jacobs, then a postdoctoral fellow at Stanford, Bill and I revisited the earlier studies of Hernandez-Peon’s group and others on cholinergic stimulation to the brain. We began experiments with microinjections of the cholinomimetic agent, carbachol, into pontine sites of cats surgically instrumented for polysomnography. Indeed, cataplectic-like episodes could be triggered with such microinjections [4]. Figure 1 comprises four excerpts of polygraph data from a cat after microinjection of carbachol in the pontine reticular formation. Our studies with cholinergic drugs at Stanford stimulated renewed interest for a number of sleep researchers using refined methods and nanogram doses at various points in the cholinergic pathways involved in REM sleep [5].

## Narcolepsy in Dogs

In 1973, while our studies with cholinergic drugs continued, Bill Dement continued to develop the sleep clinic at Stanford and to identify patients with narcolepsy. Bill, in his book, *The Promise of Sleep* [6], related the story of how he acquired for study a famous dog that displayed clinical signs of narcolepsy. The story is exciting, and reminiscent of an adventure movie. It should be read in Bill’s own prose. For the purposes of this paper, here, I include only key elements.

In 1972, at the convention of the American Medical Association, Bill showed a couple of film clips of cataplectic attacks in patients from the Stanford clinic. A neurologist approached him and told him that he knew of a dog that had similar attacks. Bill was able to contact the dog’s veterinarian and learned that the affected dog had been euthanized because of then supposed “epileptic seizures,” a film was made of the dog having the attacks. Interestingly, during this time, a number of veterinarians and breeders were describing somewhat similar postural collapses in other animals including a miniature horse [7, 8]. It appeared that these other animals were often euthanized by their owners or breeders because of clinical care expense or the breeder’s concerns about reputation or other economic reasons. Bill took serious note of the euthanized dog’s case. The next year at the American Academy of Neurology, Bill showed a film of human cataplexy and the film of the dog’s attacks. A neurologist at that meeting told Bill of a French poodle in Saskatoon, Canada that had similar symptoms. Bill was able to contact a veterinarian practicing in Saskatoon, Saskatchewan named Byron Boysen. Dr. Boysen had a case of a young female French poodle that presented to the University of Saskatchewan Veterinary Clinic in November 1972. He, along with several other veterinarians in the Saskatoon area, saw the dog clinically. It appeared to them that she was in good health except for clinical signs suggesting narcolepsy‐cataplexy. The most prominent feature was reversible, hypotonic postural collapses triggered by excitement. With the help of Bill’s Congressman and the president of the airline, restrictions against the transport of “sick animals” was overcome. The little dog, Monique, was flown out of Canada to Stanford for further study and possible breeding.

During 41 hours of continuous EEG, EOG, and EMG monitoring in conjunction with behavioral observation, the dog exhibited normal polygraphically determined wakefulness, slow-wave sleep, and REM sleep. Unambiguous sleep onset REM periods and cataplectic attacks were also documented polygraphically. These pathological manifestations were judged to be analogous to those that characterize human narcolepsy. The diagnosis of canine narcolepsy was further confirmed by two negative trials with neostigmine (ruling out myasthenia) and two positive trials with imipramine (cataplexy in human narcolepsy responds to imipramine treatment). Early publications of Monique’s case prompted referral of other dogs from various veterinary practices in North America. Thus began the Stanford narcoleptic dog colony [9].

The following years saw numerous important contributions pertaining to our narcoleptic dogs. Only a few highlights can be mentioned here.

Although the phenotypes of canine narcolepsy varied in terms of symptom severity and heritability from breed to breed, these dogs appeared to have similarities in symptoms and proved to be informative subjects for studies of drugs that could point to underlying neurochemical defects. An objective measurement was devised to measure cataplexy and changes in cataplexy after a drug, such as imipramine, was introduced.

Objective measurement of fluctuations in cataplexy was achieved with the so-called Food Elicited Cataplexy Test (FECT). Once this ad hoc test was designed, we had become familiar with these animals’ behavior and symptom triggers. The FECT entails presentation of 10, 1 cm cubes of cheese or other desired food spaced 30.5 cm apart in a row. Normal and cataplectic dogs are easily trained to eat the bits of food one after the other, while the experimenter sits at the end of the row, recording both how long it takes the dog to eat all 10 pieces, and how many full and partial cataplectic attacks occur before the last piece of food is swallowed. Normal dogs consume all the bits of food within a few seconds. Dogs with cataplexy may require several minutes and experience dozens of cataplectic episodes. Number of cataplectic episodes and elapsed time were the metrics extracted from each FECT to measure the severity of the symptom of cataplexy. Serial FECTs reflected the onset, duration, and dissipation of a drug’s anticataplectic effect. Debra Babcock and colleagues published one of the first studies using the FECT [10].

Efforts to breed dogs with narcolepsy produced a complex heritability picture. Some breeds reliably produced affected offspring (eg, Doberman pinschers). Others did not (eg, miniature poodles). Breeding efforts through 1979 were summarized by Arthur Foutz who succeeded me at Stanford’s animal sleep laboratory [11].

Several productive sleep researchers worked at the Stanford Narcoleptic Dog colony and laboratory including Tom Kilduff, Ted Baker, Seiji Nishino, and Emmanuel Mignot. Breeding and genetic studies continued and were aimed at discovering pathophysiological factors underlying canine narcolepsy. From 1976 to 1995, a total of 669 animals of various breeds were born. About 78% were Doberman pinschers [12]. A summary of the Stanford Narcoleptic Dog Colony appeared in 1998.

One study discovered an immune-like sequence that was strongly linked with the canine narcolepsy gene [13]. In 1999, Mignot’s group isolated the gene responsible for canine narcolepsy. However, this 1994 study was probably one kernel that led to Mignot’s extensive work on immune responses leading to narcolepsy [14].

In 1999 and 2000, Mignot’s group and Yanagisawa’s group published independent discoveries concerning hypocretin’s role in sleep [15, 16]. The importance of hypocretin/orexin pathways became appreciated worldwide. Nishino with Mignot’s group at Stanford showed that dogs with sporadic narcolepsy frequently have low hypocretin in CSF and brain [17]. In genetically narcoleptic Dobermans, however, hypocretin levels were not altered in brains nor in CSF, and hypocretin-containing neurons were of normal appearance, indicating a different etiology in genetically narcoleptic Dobermans. Their work seems to implicate defects in the hypocretin 2 receptor (Hcrtr 2) gene.

Indeed, a sea change took place. Narcolepsy research rapidly took new directions with manipulation of the hypocretin system. Strains of mice with murine narcolepsy were created (ie, knockout mice with defects in the CNS hypocretin system). Mignot’s group pursued more economical and efficient studies through experiments with the hypocretin system of zebra fish [18].

But those of us who studied those gentle and affectionate narcoleptic dogs will always treasure our time with them. It was a delight to work with them. Considering the breakthroughs in the understanding of sleep mechanisms that they made possible, their participation should not be forgotten.

It is a pleasure to use the opportunity offered to me by writing this paper to acknowledge the numerous scientists whose names appear with mine in the reference section. Here, I want to single-out John Orem, a Fellow in the Dement lab in the mid 1970s, and most warmly thank him for saving us all the embarrassment of having to live with my original name for our test of cataplexy severity in dogs by offering a desired food. And John did it so gracefully, too! During my progress report at one of the weekly meetings of the Dement lab on drug testing using my team’s newly devised, “Milk Bone Dog Biscuit Test,” John wryly asked me if I was really testing Milk Bone Dog Biscuits! And so, from then on, the name John suggested, “Food Elicited Cataplexy Test,” was used to measure symptom severity and response to treatment in narcoleptic dogs. This experience also reminded me of how important it is for a test’s name to be selected for its descriptive value and should, at least, point to the type of measurement involved.

## Measurement of Narcoleptic Symptoms in Humans

Contemporaneously to our sleep studies with animals, Mary Carskadon and Bill Dement maintained a vigorous program of sleep studies in children. These studies were done through the Summer Sleep Camp operating on Stanford’s Campus during the summer terms. Through the Summer Sleep Camp, Mary and Bill trained dozens of future sleep researchers. In order to learn how to conduct polysomnography in humans, I sat-in on Mary’s training sessions. A significant fraction of leading people in sleep and circadian research today were involved in Mary’s sessions. Charles Czeisler, the Frank Baldino, Jr. Professor of Sleep Medicine at Harvard and Mark Rosekind, a NASA scientist and, subsequently, the former Administrator of The National Highway Safety Administration come to mind.

One focus in Mary’s many studies was objective measurement of tendency to fall asleep and how that tendency changes over human development and over hours in the 24-hour day. Many of us spent time inspecting data from these studies, starting with unscored paper records and tabulations of various sleep parameters derived from scored records. In Mary’s papers were quantitative descriptions of changes in sleep structure over time of day and across developmental stage [19]. Mary was a frequent presenter at meetings of the Dement lab. One feature of these data that resonated with me was the waning and waxing in sleep latency data. The troughs and peaks were especially striking in the 90-minute day data [20]. Other investigators, using various measurement protocols, also found regular troughs and peaks in sleep latency and tendency for REM sleep throughout the 24-hour day [21, 22].

The rationales for studying different wake‐sleep schedules varied among investigators. Nevertheless, one collective result was a map of the temporal distribution of NREM and REM sleep throughout the 24-hour day and the characterization sleep structure on different wake‐sleep schedules that prevailed over experiments lasting for several 24-hour days. Most of these studies preserved in their protocols are the ratio of 8 hours of sleep and 16 hours of wake that is considered normal for humans. In the 90-minute day protocol, for example, the subject was permitted to sleep during a 30-minute interval every 90 minutes. Other investigators preserved the 1-to-2 ratio with different cycle lengths. The studies on this topic from Weitzman’s group offered 1 hour of sleep every 3-hour interval [21]. Lavie and colleagues used a 13-minute waking–7-minute sleep cycle [22]. All investigators, regardless of the duration of the sleep period, their protocols are offered, and reported a marked circadian rhythm in sleep tendency and the amount of REM sleep per sleep period. Also, REM sleep appeared to be linked to the 24-hour rhythm in body temperature. Carskadon et al. reported a daily pattern of occurrence of REM sleep, apparently related to the underlying body temperature fluctuation, with propensity for REM sleep to increase with the circadian rise in body temperature. These studies were valuable because they revealed fundamental characteristics of the human wakefulness-sleep system. In addition, these studies of sleep tendency throughout the 24-hour day were of interest to clinicians concerned with the evaluation of patients complaining of excessive sleepiness.

Just how abnormal was a patient’s sleep tendency? In early clinical evaluations of patients suspected of having narcolepsy, for example, a daytime nap study was often done, using some modification of a clinical EEG study. In 1960, Vogel was first to report that patients with narcolepsy had early onset of REM sleep at night on their electroencephalograms [23]. In 1963, Rechtschaffen et al [24]. and Takahashi and Jimbo [25] independently described rapid sleep onset and REM sleep periods soon after sleep onset, rather than the usual pattern of emerging after about 60-75 minutes of NREM sleep. Short sleep latency and the presence of sleep onset REM sleep began to be considered diagnostic characteristics of the sleep of patients with narcolepsy [23]. During the diagnostic workup of patients who complained of excessive sleepiness, it was of interest in sleep EEG studies whether the patient would exhibit REM sleep.

## The Multiple Sleep Latency Test

Carskadon and Dement [20] suggested that sleep latency, measured repeatedly in controlled nap situations, such as was done in the 90-minute day studies, might prove useful in the evaluation of pathological sleepiness. At the regular meetings of the Dement Lab, it was noted by several of us that the individual recording sessions involved in the 90-minute day studies collectively constituted the repeated sleep measurements suggested by Carskadon and Dement [20]. It was also noted in our laboratory meetings that each sleep recording session, especially if the sleep session was sufficiently long, could capture the emergence of REM sleep. This line of thinking led to the development of research versions and clinical versions of the Multiple Sleep Latency Test [26, 27]. The key difference between the two versions is that in the research version, sleep opportunities were terminated as soon as the subject was considered to be asleep by sleep scoring criteria. This provision was important, for example, in sleep deprivation studies, because quickly ended sleep opportunities would not allow accumulated sleep to contaminate assessment of sleep tendency. In the clinical version, sleep opportunities were a set duration (eg, 20 minutes) regardless of whether the subject falls asleep. This provision would allow time for sleep onset REM periods to emerge.

**Figure 2.**
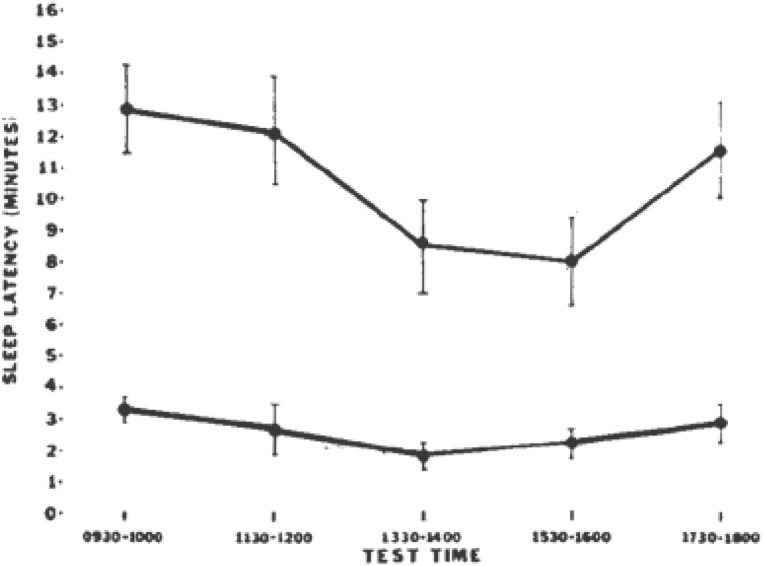
Sleep latencies in narcoleptic patients and control subjects. (reprinted from [26] with permission) Mean (±SEM) sleep latencies for control (upper curve) and narcoleptic (lower curve) subjects. For each opportunity to sleep, narcoleptics fell asleep significantly more quickly than did control subjects.

With respect to normal sleep latency, for five 20-minute-long opportunities to fall asleep offered at 2-hour intervals beginning at 10:00 o’clock am, 5 control subjects averaged sleep latencies of 11.4, 11.7, 7.0, 7.3, and 12.1 minutes. For narcolepsy, 19 patients averaged sleep latencies of 3.1, 2.9, 2.0, 2.6, and 3.2 minutes. Figure 2 is a summary graphic that combines results across several studies that called for at least five 20-minute-long opportunities to sleep offered every 2 hours at 9:30 or 10:00, 11:30 or 12:00, 13:30 or 14:00, 15:30 or 16:00, and 17:30 or 18:00. Figure 2 demonstrates that for each opportunity to sleep, narcoleptic patients fell asleep significantly more quickly than did control subjects.

In a follow-on study of REM sleep periods during daytime tests, we observed the frequency distribution of REM sleep episodes in 40 narcoleptic patients each given a Multiple Sleep Latency Test with 20-minute opportunities to sleep every 2 hours beginning at 10 am. The frequency distribution of number of REM sleep episodes was: 11 patients presented 2 REM sleep episodes, 5 presented 3, 11 presented 4, and 13 presented 5. The mean number of REM sleep episodes per subject was 3.7, SD = 1.2. By comparison, none (0) of 14 control subjects presented REM sleep episodes on the Multiple Sleep Latency Test. A repeated measures ANOVA disclosed a significant effect of testing time on sleep latency that reflected the now familiar, midafternoon drop in sleep latency. These 1978 and 1979 papers appear to be among the first to apply standardizable objective testing of sleep tendency and tendency to exhibit sleep onset REM periods in the study of patients with narcolepsy versus controls. With use and clinical experience, a set of guidelines for use of the MSLT emerged [28]. Consensus developed that an average sleep latency on the MSLT of < 5 minutes includes a range found to be associated with performance decrements and unintentional episodes of sleep in sleepy patients and in sleep-deprived nonpatient groups. The guidelines recognized that a finding of 2 or more sleep-onset REM episodes in a series of 5 sleep latency tests across a day is consistent with a diagnosis of narcolepsy. However, the guidelines noted that in patients with sleep apnea syndrome or with chronic patterns of fragmented sleep, more than 2 sleep-onset REM episodes may be observed on MSLT.

## The Maintenance of Wakefulness Test (MWT)

In some settings, the MSLT’s instruction to go to sleep was problematic for subjects who complained of falling asleep quickly at inappropriate times or who needed to stay awake for work. At Stony Brook University’s sleep disorder center, we addressed these issues by a modification of the MSLT, the Maintenance of Wakefulness Test (MWT). The MWT was designed to measure a patient’s ability to stay awake. It has been suggested that instruments like the MWT and the Repeated Test of Sustained Wakefulness measure a different aspect of the human wakefulness-sleep system than does the MSLT [29]. It has been suggested that ability to fall asleep and ability to remain awake are qualitatively different and change under different testing conditions [30]. Thus, a cousin of the MSLT was introduced, the MWT [31, 32]. Normative data for both the MSLT and the MWT have been gathered. Both tests have been standardized and are in widespread use in clinics and research laboratories throughout the world [33, 34].

Judging from reprints requests and notification of “reads” issued by ResearchGate, one of our papers that continues to be of widespread interest concerned the use of the MWT to gauge the relative efficacy of alerting drugs [35]. Working with objective data, we arithmetically averaged pretreatment sleep latencies and inspected averages of best sleep latencies observed on treatment. It was thus possible to compare the relative efficacy of putative therapeutic agents against published norms for the MSLT and MWT. As is commonly known, relative efficacy data of drugs are rarely available in sleep medicine. Most published papers on drug efficacy in the sleep literature focus on a single drug and report on that drug vs. placebo contrasts for various parameters, for example, total sleep time, sleep latency, etc. The MWT and the normative database for the MWT permit inter-drug comparisons of a conceptually different nature. This may be one explanation of the interest in our relative efficacy paper.

## Sleep Tendency Detection Throughout the 24 Hours

**Figure 3.**
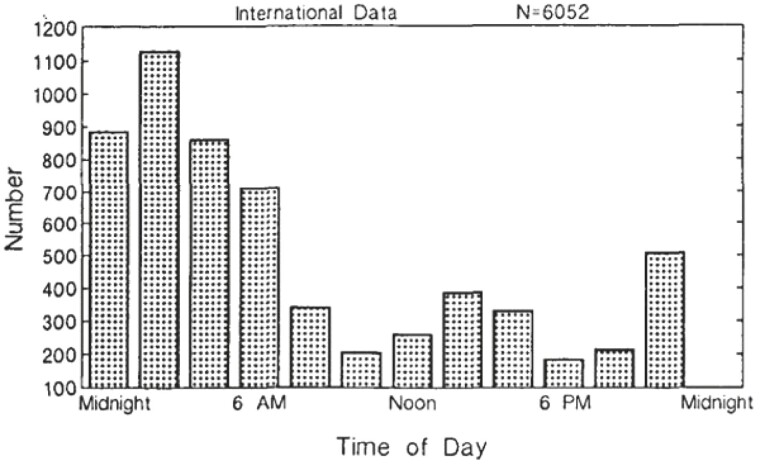
Vehicle crashes versus time of day (reprinted from [40, 41], with permission). The figure combines the samples of [40, 41] (Texas: *n* = 4994) and Duff (unpublished observations) (New York: *n* = 668).

As was mentioned earlier, data from the 90-Minute Day studies contained objective measurement of sleep tendency through the 24 hours. We have inspected 24-hour data sets gathered for reasons pertaining to Federal regulation and public policy. Data from studies of human performance during the workday revealed 24-hour patterns that suggested 24-hour performance data contained signals from the human wakefulness–sleep cycle. For us, one of the most striking signals was the afternoon peak in performance errors [36]. [NB: Throughout this paper, it should be remembered that daily rises (peaks) in sleep tendency are reflected in decreases in measures of sleep latency and concomitant rises in performance errors.] We found that this mid-afternoon signal was detectable in 24-hour patterns of very different parameters, including vehicle crashes and disease-related mortality [37–39]. As shown in Figure 3, the frequency distribution of 6052 vehicle crashes judged by investigators to have been related by operator fatigue, early morning and mid-afternoon frequency peaks are visible.

**Figure 4.**
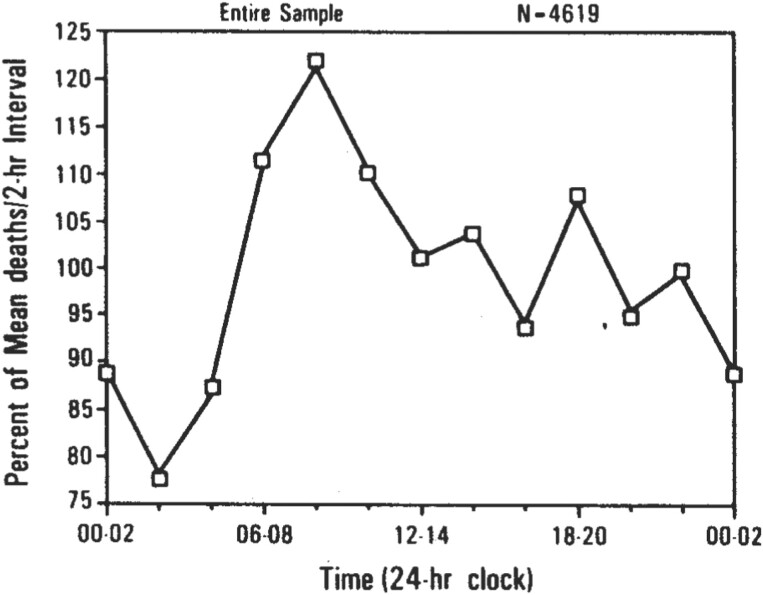
Deaths versus time of day (reprinted from [39] with permission). The temporal distribution of death for 4619 disease-related deaths read from death certificates for disease-related deaths (ie, deaths not involving trauma) that occurred in New York City during the year 1979. The horizontal axis is successive 2-hour intervals throughout the day. There were 2350 males (mean age: 70.73; standard deviation: 13.9) and 2269 females (mean age: 74.75; standard deviation: 13.2). The temporal distribution of deaths for the entire sample is presented in the figure. Reported mortality during the 8 am to 10 am (08:00 to 10:00) interval was 60% greater than during the 2 am to 4 am (02:00 to 04:00) interval. The overall effect of time of day was highly significant (*P* < 0.001) and was confirmed in the separate replications for males (*P* < 0.001) and females (*P* < 0.01). Note also the smaller, late afternoon peak during the 6 pm to 8 pm (18:00 to 20:00) interval.

There are also morning and afternoon peaks in disease-related death. Figure 4 presents disease-related deaths versus time of day. The temporal distribution of death for 4619 disease-related deaths read from death certificates for disease-related deaths (ie, deaths not involving trauma) that occurred in New York City during the year 1979. The horizontal axis is successive 2-hour intervals throughout the day. There were 2350 males (mean age: 70.73; SD: 13.9) and 2269 females (mean age: 74.75; SD: 13.2). The temporal distribution of deaths for the entire sample is presented in the figure. Reported mortality during the 8 am to 10 am (08:00 to 10:00) interval was 60% greater during the 2 am to 4 am (02:00 to 04:00) interval. The overall effect of time of day was highly significant (*P* < 0.001) and was confirmed in the separate replications for males (*P* < 0.001) and females (*P* < 0.01). Note also the smaller, late afternoon peak during the 6 pm to 8 pm (18:00 to 20:00) interval.

James Miller and I devised a mathematical model that was adjusted to fit the 2-peak patterns observed in vehicle crashes and disease-related mortality [36, 37]. In the 1996 paper, we fitted a 2-peak-per-day cosine curve derived from the population growth function used in chaos theory. The model was intended to be heuristic, not explanatory. We acknowledged that the temporal associations described in our paper do not imply causation. For example, pathophysiological explanations are not presently in hand for exactly why an afternoon increase in sleep tendency should be associated with or cause an increased risk of heart attack. One could, perhaps, think of such physiological factors as increased upper airway resistance from apnea events during afternoon naps leading to stroke or heart attack. In any case, the point I wish to make here is that temporal association between events cannot be confused with causation.

**Figure 5.**
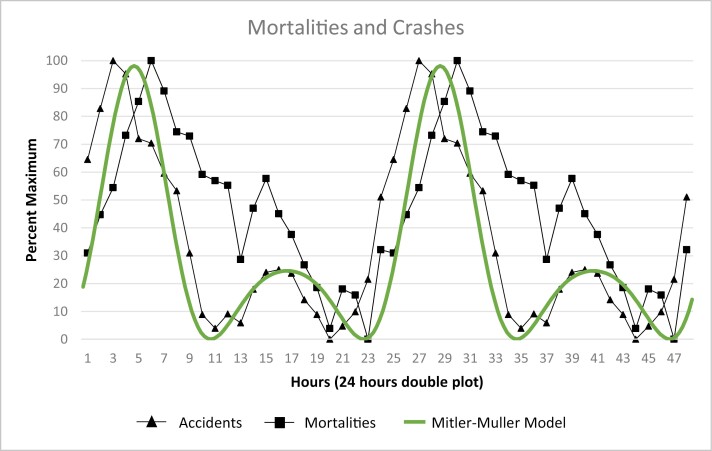
Superimposition of mortalities and vehicles crashes on the Mitler Miller model (redrawn from [37]). Double plots of the 2-peak mathematical model shown co-plotted with human mortality data and vehicle crash data. Depicted is the fit of our 2-peak model (smooth curve) with 2 large data sets: (a) MORTALITIES: temporal distribution of disease-related deaths comprised from the work of Michael Smolensky’s group [42]. and our group [39] and, (b) ACCIDENTS: 6052 vehicle crashes from a composite of data sets of Lavie from Israel [40], Langlois et al. from Texas, USA [41], and Duff (personal communication, 1990) from New York. The model’s agreement with the 2 data sets was highly statistically significant (all *P*s < .01).

Figure 5 depicts the fit of our 2-peak model (smooth curve) with 2 large data sets: (a) Mortalities: temporal distribution of disease-related deaths comprised from the work of Michael Smolensky’s group [42] and our group [39] and (b) Accidents: 6052 vehicle crashes from a composite of data sets of Lavie from Israel [40], Langlois et al. from Texas, USA [41], and W. Duff (personal communication, 1990) from New York. The model’s agreement with the 2 data sets was highly statistically significant (all *P*’s < .01).

Other investigators continue to study this 2-peak pattern. The theoretical and experimental work of Alexandre Borbély [43] will continue to be useful in understanding afternoon peaks in sleep tendency. His 2-process model attributes the afternoon peak as a phenomenon stemming from circadian factors interacting with increasing sleep pressure from the sleep homeostat as daytime wakefulness continues through the day.

Articles cited in this section do seem to have stimulated research on better understanding of why there is such a persistent temporal linkage between 24-hour sleep patterns and pathological events such as heart attack and stroke.

Also, ideas from the many papers reporting on the 2-peak pattern in man’s alertness throughout the 24-hour day have had some beneficial effect on public policy. Regulatory rulings in transportation and physician training have begun to reflect, at least to some degree, the vulnerability of humans to fatigue-related errors in the early morning of the 24-hour day. For example, Hours of Service (HOS) recommendation for interstate truck drivers have been revised several times in recognition of the increased tendency of truck drivers to make errors during the early morning hours (https://www.fmcsa.dot.gov/regulations) [44, 45].

## Sleep Disorders Research and Government Affairs

Some readers may know that I have had a lifelong interest in the organization and workings of our government in Washington, DC. I had been active in the government affairs committees of the sleep medicine and research communities. All of us were gratified when The National Center on Sleep Disorders Research (NCSDR) was finally established in 1993. Efforts toward this center had been ongoing for years, beginning with testimonies before Congressional Committees by myself and other sleep researchers, attending policy forums, etc. These efforts were continuously championed by Bill Dement with support from the leadership of the SRS and the AASM.

I was fascinated working throughout the lengthy process of meetings with Members of Congress, NIH leadership, and our government affairs consultants, Dale Dirks and his Health and Medicine Counsel of Washington. The NCSDR ended-up being administratively housed in the National Heart, Lung, and Blood Institute. The Director of NHLBI at that time was Dr. Claude Lenfant, a prominent pulmonary physician who had been influential in academic and government circles for decades. Through many months of deliberations with Congressional staff, leadership of the sleep research communities and NHLBI, the NCSDR was structured so that all NIH Institutes and Centers (ICs) could be represented. The ICs that supported a great many NIH-funded extramural projects were given permanent representation at NCSDR meetings and activities.

While having the NCSDR in place was an important step forward for sleep disorders research, it soon became apparent that a great deal more work was needed to secure sleep disorders research a stable position among competing specialties. The field of sleep disorders research had become more visible. But with that, came more competition for recognition and resources throughout the NIH. The presence of the NCSDR did not automatically make it easier for sleep researchers to get their NIH grant applications funded. Competition with other specialty fields was, as always, intense. An important piece of advice the sleep research leadership was getting from NIH officials who had previously seen other new fields grow and flourish was to train more sleep disorders investigators, submit more competitive grant applications and better align grant applications with the standing funding priorities of the NIH ICs. I believe that the SRS and the AASM followed this advice and continue to work in these directions.

In 2002, I was recruited to join the National Institute on Neurological Disorders and Stroke (NINDS) at the NIH in Washington. The NINDS is the institute that supported most of my academic research for many years. I seemed to have the appropriate background and research experience for the job. And I was interested in helping build on what was achieved by the formation of the National Center on Sleep Disorders Research. So, Elizabeth and I left beautiful San Diego, California for Bethesda, Maryland.

I remember the transition from the private to the public sector to be exciting, but also bittersweet. My NIH position required that I pass on to others my role at Scripps of Principal Investigator on government grants and contracts. My new job was to programmatically review the grant applications of others and assume the duties of an NIH Program Officer for funded NIH research projects. Soon, I became immersed in day-to-day administration of NINDS grants and contracts. I was one of hundreds of Federal officials in the development of research programs in various areas of biomedical science. I had viewed sleep medicine as a component of mainstream medical science. I worked to help others in the Federal government to see this too. I worked at NINDS within the NIH for about ten and a half years (12/19/2002 to 6/30/2013).

My assignment at NINDS was to administer grants and contracts pertaining to Systems and Cognitive Neuroscience. These awards included sleep and circadian rhythms research. My duties often involved explaining to sleep researchers who held NIH grants why the National Center was placed in NHLBI (Heart, Lung, and Blood) and not in some other NIH institute such as NIMH (Mental Health) or NIGMS (General Medical Sciences)‐not always an easily understood explanation. Nevertheless, by the end of my government service, I think that it was safe to say that the concepts sleep and circadian rhythms, including the notion of time-dependent changes in disease, symptom severity, and sensitivity to therapeutics, had become recognized throughout the Federal government and medicine world-wide https://nigms.nih.gov/education/fact-sheets/Pages/circadian-rhythms.aspx. The NIH Sleep Research Plan Strategic Goals for 2021 formulated by the National Center on Sleep Disorders Research seem to speak for themselves.

Goal 1: Sleep and Circadian Mechanisms Underlying Health and Disease
Goal 2: Risk Reduction and Treatment of Sleep and Circadian Disorders
Goal 3: Clinical Implementation of Sleep and Circadian Research
Goal 4: Sleep and Circadian Disruptions and Health Disparities
Goal 5: Sleep and Circadian Biology Research Workforce Development

For me, 2 events come to mind as significant markers in the continuing progress of the field of sleep disorders research. One is the 2017 Nobel Prize in Medicine awarded to Drs. Michael Young, Michael Rosbash, and Jeffery Hall for their discoveries of molecular mechanisms underlying circadian rhythms [46]. Much of Michael Young’s work was supported by NINDS. The work of these three scientists established, over a period of years in the 1980s and 1990s, our present understanding of the interaction between the Drosophila genes, *timeless* (tim) and *period* (per). This understanding unfolded over years with the efforts of numerous scientists well-known to the sleep research community including such scientists as Amita Sehgal and Charles Weitz.

The second important marker for the field, and the last I will comment on here, was the awarding of the 2023 Breakthrough Prize in Life Sciences to Emmanuel Mignot and Masashi Yanagisawa for their independent discoveries on hypocretin/orexin in the etiology of narcolepsy. [see: Breakthrough Prize]. Mignot’s research, supported in part by grants from NINDS, identified in narcoleptic dogs that their sleep disorder was due to a mutated gene that belonged to a cell membrane receptor for a molecule that was subsequently identified as hypocretin. Yanagisawa and Dr. Sakurai at the University of Tsukuba, Japan, had been working on feeding control. They discovered orexins in 1999 thinking that these molecules were appetite inducing (thus the name “orexin”). They knocked out the orexin gene in mice and subsequently found that the orexin-deficient mice seemed to transition rapidly in REM sleep, displaying symptoms of narcolepsy. Yanagisawa found mice lacking the orexin gene were no longer nocturnal and instead displayed sleep attacks at night. A comparison of data led to the present understanding that Yanagisawa’s and Mignot’s groups were both dealing with the same molecule. Deficiency of this molecule, termed, “orexin,” by some investigators and “hypocretin” by others, produced narcolepsy. To minimize confusion, people often refer to the molecule as orexin/hypocretin. Mignot theorized that narcolepsy is caused by an autoimmune disorder that selectively destroys CNS neurons that produce orexin/hypocretin. This thinking fits with previously mentioned work by Mignot’s group implicating autoimmune responses in the etiology of narcolepsy. I appreciate that Emmanuel will soon be writing his own paper for this journal. But since Emmanuel and I both spent so much time studying narcolepsy, I hope my few words here will not detract, but, instead, what the appetite for his paper. Well, I also realize that I need to make an end to the present paper, somehow.

In closing, I want to thank the editors of *SLEEP Advances* for this opportunity to look back and offer my perspectives. It has been an honor and a pleasure. I am sorry that for reasons of journal space, I could not describe the substantive input received from the many prominent scientists who worked in the various facilities or visited me over the years. Among these people, just to name a few, were Christian Guilleminault, Vincent Zarcone, Elliot Weitzman, Daniel Kripke, Tom Roth, Michel Jouvet, Allan Rechtschaffen, and Ismet Karacan. I see that many on this list are, sadly, no longer with us. To those who are and to the Editors of *SLEEP Advances* thank you for this chance to look back and reflect. I hope these reminiscences did not put you to sleep. But sleep researchers know that they can never admit to such a thing. Falling asleep while reading does not indicate boredom, it indicates the presence of a sleep disorder.
